# Iron affects the sphere-forming ability of ovarian cancer cells in non-adherent culture conditions

**DOI:** 10.3389/fcell.2023.1272667

**Published:** 2023-11-14

**Authors:** Anna Martina Battaglia, Alessandro Sacco, Eleonora Vecchio, Stefania Scicchitano, Lavinia Petriaggi, Emanuele Giorgio, Stefania Bulotta, Sonia Levi, Concetta Maria Faniello, Flavia Biamonte, Francesco Costanzo

**Affiliations:** ^1^ Laboratory of Biochemistry and Cellular Biology, Department of Experimental and Clinical Medicine, Magna Graecia University of Catanzaro, Catanzaro, Italy; ^2^ Laboratory of Biochemistry and Biology, Department of Health Sciences, Magna Graecia University of Catanzaro, Catanzaro, Italy; ^3^ Vita-Salute San Raffaele University and San Raffaele Scientific Institute, Milan, Italy; ^4^ Department of Experimental and Clinical Medicine, Center of Interdepartmental Services (CIS), Magna Graecia University of Catanzaro, Catanzaro, Italy

**Keywords:** iron metabolism, ovarian cancer, TME, tumor spheroids, ECM detachment, FtH1, ferroptosis

## Abstract

**Introduction:** Detachment from the extracellular matrix (ECM) is the first step of the metastatic cascade. It is a regulated process involving interaction between tumor cells and tumor microenvironment (TME). Iron is a key micronutrient within the TME. Here, we explored the role of iron in the ability of ovarian cancer cells to successfully detach from the ECM.

**Methods:** HEY and PEO1 ovarian cancer cells were grown in 3D conditions. To mimic an iron rich TME, culture media were supplemented with 100 μM Fe^3+^. Cell mortality was evaluated by cytofluorimetric assay. The invasive potential of tumor spheroids was performed in Matrigel and documented with images and time-lapses. Iron metabolism was assessed by analyzing the expression of CD71 and FtH1, and by quantifying the intracellular labile iron pool (LIP) through Calcein-AM cytofluorimetric assay. Ferroptosis was assessed by quantifying mitochondrial reactive oxygen species (ROS) and lipid peroxidation through MitoSOX and BODIPY-C11 cytofluorimetric assays, respectively. Ferroptosis markers GPX4 and VDAC2 were measured by Western blot. *FtH1* knockdown was performed by using siRNA.

**Results:** To generate spheroids, HEY and PEO1 cells prevent LIP accumulation by upregulating FtH1. 3D HEY moderately increases FtH1, and LIP is only slightly reduced. 3D PEO1upregulate FtH1 and LIP results significantly diminished. HEY tumor spheroids prevent iron import downregulating CD71, while PEO1 cells strongly enhance it. Intracellular ROS drop down during the 2D to 3D transition in both cell lines, but more significantly in PEO1 cells. Upon iron supplementation, PEO1 cells continue to enhance CD71 and FtH1 without accumulating the LIP and ROS and do not undergo ferroptosis. HEY, instead, accumulate LIP, undergo ferroptosis and attenuate their sphere-forming ability and invasiveness. *FtH1* knockdown significantly reduces the generation of PEO1 tumor spheroids, although without sensitizing them to ferroptosis.

**Discussion:** Iron metabolism reprogramming is a key event in the tumor spheroid generation of ovarian cancer cells. An iron-rich environment impairs the sphere-forming ability and causes cell death only in ferroptosis sensitive cells. A better understanding of ferroptosis sensitivity could be useful to develop effective treatments to kill ECM-detached ovarian cancer cells.

## 1 Introduction

Iron plays a key role in all the steps of tumorigenesis (Guo et al., 2021; Salnikow, 2021). Cancer cells, as a result of their increased proliferative rate and enhanced metabolic activity, usually show a pronounced iron addiction (Rodriguez et al., 2022; Battaglia et al., 2023). To achieve the iron demand, tumor cells overexpress proteins involved in iron intake (i.e., transferrin receptor, CD71) and reduce expression of those involved in iron export (i.e., ferroportin, FPN). At the same time, tumor cells strictly regulate the expression of ferritin, protein made up of ferritin heavy chain (FtH1), provided with ferroxidase activity, and ferritin light chain (FTL), to properly store the intracellular free iron and, thus, control its participation in the Fenton reactions-mediated production of reactive oxygen species (ROS) (Pfeifhofer-Obermair et al., 2018; Morales and Xue, 2021; Sacco et al., 2021). The tumor microenvironment (TME) is a major source of iron (Liang and Ferrara, 2021). Both resident and recruited cells within the TME, such as tumor-associated macrophages (TAMs) and cancer-associated fibroblasts (CAFs), may release iron in the surrounding niche, thus creating the perfect storm for further ROS generation (Brown et al., 2020; Biamonte et al., 2021a; Sacco et al., 2021; Tamariz-Amador et al., 2021). When accumulated at moderate levels, ROS may promote widespread modifications of DNA, proteins, and lipids that, overall, lead to a more aggressive tumor phenotype (Brown et al., 2020). ROS can also induce metabolic rewiring toward the so-called “Warburg effect” characterized by the glycolysis-mediated overproduction and release of lactic acid. Lactate accumulation creates an acidic TME, which breaks down the extracellular matrix (ECM) through the activation of matrix metalloproteinases (MMPs) (Pérez-Tomás and Pérez-Guillén, 2020; Niland et al., 2022). Iron and iron-dependent ROS may activate epithelial-to-mesenchymal transition (EMT) through the regulation of the CXCR4 and Wnt/β-catenin signaling pathways, as well as through the regulation of a panel of oncomiRNAs, thus promoting tumor cells migration and invasion (Biamonte et al., 2015; 2018; Aversa et al., 2017). In addition, ROS-mediated hypoxic conditions promote neo-vascularization through the activation of vascular endothelial growth factor (VEGF) pathway (Kim and Byzova, 2014). In this way, iron alters the behavior of tumor cells and shapes the local TME to successfully complete all the stages of the metastatic cascade (Brown et al., 2020). A pronounced iron demand has, though, another side of the coin. When intracellular free iron accumulates beyond the storage capacity of FtH1, the massive production of ROS overwhelms the intracellular antioxidant defenses and causes ferroptosis (Battaglia et al., 2020; 2022; Di Sanzo et al., 2020). Ferroptosis is a regulated cell death (RCD) presenting unique features such as the presence of oxidizable phospholipids acylated with polyunsaturated fatty acids (PUFA-PLs), defective or inhibited lipid peroxide repair mechanisms, and mitochondrial dysfunction (Dixon and Stockwell, 2019). We and others have demonstrated that vulnerability to ferroptosis significantly varies among tumor cells and depends both on intrinsic and extrinsic factors (Sacco et al., 2021; Battaglia et al., 2022; Zhang et al., 2022; Chen et al., 2023). The extent of “iron-addicted phenotype” is determined by the innate expression pattern of iron regulatory proteins (i.e., CD71, FtH1, and FPN) and by the relative innate intracellular iron levels, and ultimately affects the sensitivity of tumor cells to ferroptosis (Rodriguez et al., 2022). Furthermore, an iron-rich TME derived either from high-iron diets or iron treatments may sensitize cells to ferroptotic cell death (Chen et al., 2020).

Metastasis is a major contributor to cancer mortality (Seyfried and Huysentruyt, 2013). To successfully metastasize to a secondary site, tumor cells must detach from the extracellular matrix (ECM) (Elgundi et al., 2019). Only a small number of tumor cells, the so-called “persister cancer cells” with a stem cell-like phenotype, adapt to survive in non-adherent conditions (Rodriguez et al., 2022); the vast majority of tumor cells, instead, succumb to ECM-detachment and face cell death (Buchheit et al., 2012). The specific mechanisms through which ECM-detached cells are eliminated remain incompletely understood. Anoikis, the caspase-dependent apoptotic cell death caused by the loss of integrin-mediated adhesion to ECM, has been considered for a long time as the unique cell death modality associated with ECM-detachment (Vachon, 2011; Adeshakin et al., 2021). Recently, though, a number of reports have demonstrated that the sole inhibition of anoikis is not sufficient to guarantee the long-term viability of ECM-detached cells (Mason et al., 2017; Hawk and Schafer, 2018b; Qiu et al., 2022), thus suggesting that anoikis is unlikely to be the only event that suppresses tumor cells survival in ECM-detached conditions. In response to ECM detachment, a plethora of biochemical alterations leads to the robust increase in damaging ROS that overall inhibit fatty acid oxidation (FAO), thus leading to a bioenergetic crisis that causes cell death (Mason et al., 2017). Persister cancer cells hijack their metabolic routes for neutralizing oxidative stress essentially by intensifying glucose uptake and diverting glycolytic intermediates into the Pentose Phosphate Pathway (PPP), which results in the accumulation of NADPH and ROS neutralization (Ghanbari Movahed et al., 2019; Tang et al., 2022). Alternatively, persister cancer cells intensify their army of antioxidant enzymes (Cockfield and Schafer, 2019). In particular, nuclear-factor erythroid 2-related factor2 (NFE2L2/Nrf2), a master transcription factor of antioxidant genes (i.e., catalases and superoxide dismutases), has been found significantly elevated in ECM-detached cells (Endo et al., 2020; Hiebert, 2021). Dietary supplementation of antioxidants or genetic overexpression of antioxidant enzymes in animal models of lung cancer and melanoma resulted in enhanced distant metastasis (Cockfield and Schafer, 2019). Conversely, the knockdown of a single antioxidant enzyme (either catalase or superoxide dismutase) is able to efficiently compromise the viability of ECM-detached cells and anchorage-independent growth both *in vitro* and *in vivo*.

An emerging intriguing avenue under investigation indicates that the massive accumulation of ROS during ECM detachment activates ferroptosis (Hawk and Schafer, 2018a; He et al., 2023). Just this year, (He et al., 2023) uncovered a direct link between ECM detachment and iron metabolism. They demonstrate that iron uptake and iron storage are altered during ECM detachment in order to minimize the intracellular levels of free (redox-active) iron. This iron metabolic reprogramming makes ECM-detached cells resistant to ferroptosis. These data raise the possibility that iron metabolism could be targeted in a fashion that specifically eliminates cells during ECM detachment.

In this study, we analyze the reprogramming of iron metabolism of HEY and PEO1 ovarian cancer cell lines during the transition from adherent (2D) to non-adherent (3D) culture conditions. Epithelial ovarian cancer (EOC) is an aggressive disease with a still bad prognosis due to the high metastatic potential. For this reason, there is a compelling need to shed light on the intracellular molecular mechanisms and the environmental cues that cooperate to promote tumor cell survival in ECM-detached conditions. Overall, our results indicate that ovarian cancer cell growth in non-adherent culture conditions requires a modification of iron metabolism essentially aimed at preventing the accumulation of free and redox-active iron through the upregulation of its storage protein FtH1. Excess environmental iron or FtH1 knockdown are sufficient to impair the sphere-forming ability of both HEY and PEO1 cells. However, this is accompanied by a significant mortality through ferroptosis only in HEY detached cells. These data suggest that the diverse intercellular ferroptosis sensitivity might constitute a significant determinant in the success of ovarian cancer cells to detach from ECM and drive metastasis.

## 2 Materials and methods

### 2.1 Cell lines and cell culture

Human epithelial ovarian cancer (OVCA) cell lines HEY and PEO1 were purchased from the American Type Culture Collection (ATCC, Rockville, MD, United States). Following ATCC instruction, HEY cells were grown in DMEM medium (Sigma-Aldrich, St. Louis, Missouri, United States ), while PEO1 cells were cultured in RPMI 1640 (Sigma-Aldrich, St. Louis, Missouri, United States), both supplemented with 10% (v/v) fetal bovine serum (FBS) (Invitrogen, San Diego, CA), L-glutamine and 1% (v/v) penicillin and streptomycin (Sigma-Aldrich, St. Louis, Missouri, United States) at 37°C in a humidified incubator with 5% CO_2_ atmosphere. All the cell lines were tested for *mycoplasma* contaminations and STR profiled for authentication.

3D tumor spheroids were grown in a customized spheroid medium, consisting of DMEM/F-12 (Sigma-Aldrich, St. Louis, Missouri, United States) supplemented with 0.5% Glucose (Sigma-Aldrich, St. Louis, Missouri, United States), 2.5 mM L-Glutamine (Thermo Fisher Scientific, Waltham, Massachusetts, United States), 2% B-27 (Sigma-Aldrich, St. Louis, Missouri, United States), 5 μg/mL Heparin (Sigma-Aldrich, St. Louis, Missouri, United States), 20 μg/mL Insulin (Thermo Fisher Scientific, Waltham, Massachusetts, United States), 20 ng/mL EGF (Thermo Fisher Scientific, Waltham, Massachusetts, United States), 20 ng/mL Recombinant Human bFGF (Thermo Fisher Scientific, Waltham, Massachusetts, United States), 0.1% Bovine Serum Albumin (BSA) (Sigma-Aldrich, St. Louis, Missouri, United States), and 1% (v/v) of penicillin/streptomycin 100 U/mL. Briefly, 15.000 cells/mL were resuspended in an appropriate amount of medium and seeded into ultra-low attachment (ULA) plates (Corning Inc., New York, United States) to form 3D spheroids. As previously described (De Vitis et al., 2023), after 4 days, the collected tumor spheroids were resuspended in appropriate volume of culture medium and counted using the Leica THUNDER Imaging Systems DMi8 (Leica Microsystems S.r.l., Wetzlar, Germany) according to the following formulas:

$$sphere\;concentration=sphere\;count÷counting\;volume\;\left(\mu L\right)$$

$$total\;sphere\;count=sphere\;concentration\times total\;volume\;\left(\mu L\right)$$

Their diameters were then measured using the internal measuring feature of Zen imaging software (Leica Camera AG, Wetzlar, Germany, Europe) and normalized to 100 3D tumor spheres. To obtain single cell suspension that can be manipulated and stained similarly to 2D cultures to perform flow cytometry analysis, spheroids were harvested, rinsed PBS (Sigma-Aldrich, St. Louis, Missouri, United States), and incubated at 37°C for 10 min with StemProTM Accutase™ Cell Dissociation Reagent (Thermo Fisher Scientific, Waltham, Massachusetts, United States) followed by a gentle mechanical dissociation.

### 2.2 Reagents

Ferlixit (62.5 mg/5 mL, sodium ferric gluconate complex in sucrose, SANOFI) has been obtained from the outpatient pharmacy at the Unit of Cardiology, “Magna Graecia” University of Catanzaro. Ferlixit was used at the final concentration of 100 µM. Treatments were performed at least three times on independent biological replicates.

### 2.3 Measurement of the labile iron pool (LIP)

Intracellular labile iron concentration was determined by flow cytometry using the fluorescent iron sensor calcein acetoxymethyl ester (CA-AM). Briefly, single cells derived from both 2D, and 3D cultures were incubated with 0.25 μM CA-AM (Aldrich, Missouri, United States) for 30 min at 37°C in the dark. Then, cells were washed twice with PBS to remove the excess of CA-AM, and thus treated with 200 μM of the iron chelator L1 (3-Hydroxy-1,2-dimethyl-4(1H)-pyridone, Sigma-Aldrich, Missouri, United States) or left untreated. The analysis was performed by FACS BD LSRFortessa™ X-20 cytofluorometer (BD Biosciences). The difference in cellular fluorescence after and before incubation with L1 reflected the labile iron pool:

$$\Delta Mean\;Fluorescence\;Intensity,\Delta MFI={\Delta MFI}^{after}-{\Delta MFI}^{before}$$

### 2.4 Measurement of intracellular ROS

Intracellular ROS amounts were determined by incubating cells from both 2D, and 3D cultures for 10 min at 37°C with the redox-sensitive probe 2′-7′-Dichlorodihydrofluorescein diacetate (CM-H2DCFDA; Thermo Fisher Scientific, Waltham, United States), according to the instructions of the manufacturer. CM-H2DCFDA fluorescence was analyzed by flow cytometry using a FACS BD LSRFortessa™ X-20 cytofluorometer (BD Biosciences) and data were processed with FlowJo software (Tree Star, Inc.). Each experiment was performed in triplicate.

### 2.5 PI staining analysis

Single cell suspensions derived from both 2D, and 3D cultures were centrifuged and incubated with PI staining in the dark at 37°C for 15 min. Samples were then washed twice with PBS. Flow cytometry assay was performed using the BD LSRFortessa™ X-20 (BD Biosciences, San Jose, CA, United States). A total of 2×10^4^ events were acquired for each sample. Data analysis was carried out using FlowJo™ v10 Software (BD Biosciences, San Jose, CA). Each experiment was performed in triplicate.

### 2.6 Mitochondrial ROS analysis

The generation of mitochondrial ROS was measured by flow cytometry with the use of the MitoSOX Red Mitochondrial Superoxide Indicator (Thermo Fisher Scientific, Waltham, Massachusetts, United States). Upon ferlixit treatment, single cell suspensions derived from 3D tumor spheroids disaggregation were incubated with 5 µM MitoSOX for 10 min at 37°C and then analyzed by using the BD LSRFortessa™ X-20 (BD Biosciences, San Jose, CA, United States). A minimum of 20.000 cells was analyzed per condition. Fluorescence was measured using the FlowJo™ software (Tree Star Inc., Ashland, Oregon, United States). Each experiment was performed in triplicate.

### 2.7 Lipid peroxidation analyses (BODIPY-C11)

Lipid peroxidation was investigated through flow cytometry using BODIPY™ 581/591C11 dye (Thermo Fisher Scientific, Waltham, United States). Briefly, 3D tumor spheroids derived single cells were incubated at 37°C for 30 min with 2.5 µM BODIPY™ 581/591 C11; unincorporated dye was removed by washing twice with PBS. Oxidation of BODIPY-C11 resulted in a shift of the fluorescence emission peak from ∼590 nm to ∼510 nm proportional to lipid ROS generation. Flow cytometry assay was performed using the BD LSRFortessa™ X-20 (BD Biosciences, San Jose, CA, United States). A minimum of 20.000 cells was analyzed per condition. Data analysis was carried out using FlowJo™ v10 Software (BD Biosciences, San Jose, CA). Each experiment was performed in triplicate.

### 2.8 Total protein extraction

Total protein extracts from both 2D and 3D cultures were obtained using RIPA buffer containing 1 M Tris HCl, Triton X-100, 3 M NaCl, 0.5 M EDTA, 10% SDS supplemented with cOmplete™ Protease Inhibitor Cocktail provided in EASYpacks (Roche Diagnostics, Mannheim, Germany). Briefly, cells were lysed in ice-cold RIPA buffer and, after removal of the cell insoluble fragments through centrifugation at 12.000 *g* for 30 min at 4°C, protein content was quantified by Bio-Rad Protein Assay Dye according to manufacturer’s instructions (Bio-Rad Laboratories, Hercules, California, United States).

### 2.9 Western blotting

Each protein sample (40–50 µg) was separated by using 10%–15% SDS-PAGE and then transferred to nitrocellulose membranes (Sigma-Aldrich, St. Louis, Missouri, United States). After blocking with 5% milk, incubation with primary antibody was performed overnight at 4°C. The antibody against FtH1 (1:200, sc-376594) was purchased from Santa Cruz Biotechnology (Cruz Biotechnology, Dallas, Texas, United States); antibodies against voltage-dependent anion channel 2 (VDAC2) (1:500, ab37985), glutathione peroxidase 4 (GPX4) (1:1,000, ab19534), and FPN (1:500, ab235166) were purchased from Abcam (Abcam, Cambridge, UK), while antibody against CD71 (1:1,000, #13208) was obtained from Cell Signaling Technology (Danvers, Massachusetts, United States). After incubation with peroxidase-conjugated secondary antibodies (Peroxidase AffiniPure Sheep Anti-Mouse IgG, 1:10,000; Peroxidase AffiniPure Donkey Anti-Rabbit IgG, 1:10,000; Peroxidase AffiniPure Donkey Anti-Goat IgG, 1:10,000; Jackson ImmunoResearch Europe Ltd., Cambridge, UK) for 1 h at room temperature, signals were detected using chemiluminescence reagents (ECL Western blotting detection system, Santa Cruz Biotechnology, Dallas, Texas) and acquired by Uvitec Alliance Mini HD9 (Uvitec Cambridge, UK). To calculate the relative expression of specific protein, a goat polyclonal anti-γ-Tubulin antibody (γ-TUB, 1:3,000; sc-17787; Santa Cruz Biotechnology) serves as a reference for sample loading. The protein band intensity on western blots was quantified and normalized to that of γ-TUB by using ImageJ software (http://rsb.info.nih.gov/ij/).

### 2.10 *FtH1* transient knockdown of 3D tumor spheroids

2D PEO1 cells were transfected using Lipofectamine™ 3,000 Transfection Reagent (Thermo Fisher Scientific, Waltham, MA, United States) according to the manufacturer’s protocol. *FtH1* siRNA was purchased from Thermo Fisher Scientific. To ensure an optimal control, cells were further transfected with Silencer™ Select Negative Control siRNA (ctrl) (Thermo Fisher Scientific, Waltham, MA, United States). Briefly, after 8 h of transfection, 2D PEO1 cells were trypsinized and collected to generate 3D tumor spheroids for 96 h. The transfection efficiency in 3D tumor spheroids was evaluated by using qRT-PCR.

### 2.11 RNA isolation and comparative qRT-PCR analysis

Total RNA was extracted using the Trizol RNA isolation method (Life Technologies, Carlsbad, California, United States) as previously described (Di Sanzo et al., 2016; Biamonte et al., 2017; Zolea et al., 2017; Biamonte et al., 2021b). All samples were DNase treated (Thermo Fisher Scientific, Waltham, Massachusetts, United States) and purity/integrity check was performed spectroscopically before use. Then, 1 µg of total RNA was retrotranscribed using Applied Biosystems™ High-Capacity cDNA Reverse Transcription Kit (Thermo Fisher Scientific, Waltham, Massachusetts, United States). qRT-PCR was performed using the SYBR™ Green qPCR Master Mix (Thermo Fisher Scientific, Waltham, Massachusetts, United States). Analysis was performed on Applied Biosystems™ QuantStudio™ 3 (Thermo Fisher Scientific). The relative mRNA expression level of *OCT4*, *NANOG*, *SOX2*, *E-CAD*, *VIM*, *SNAIL*, *SLUG* and *FtH1* was calculated through the 2^−ΔΔCT^ method and glyceraldehyde 3-phosphate dehydrogenase (GAPDH) was used as the housekeeping gene. Each experiment was performed in triplicate. Primers used for qRT-PCR are as follows: *FTH1* (fw: 5′-ttg​acc​gag​atg​atg​tgg​ct-3′, rev: 5′-cca​gtt​tgt​gca​gtt​cca​gt-3′); *GADPH* (fw: 5′-caa​att​cca​tgg​cac​cgt​ca-3′, rev: 5′-ggc​aga​gat​gat​gac​cct​tt-3′); *OCT4* (fw: 5′-ttc​agc​caa​acg​acc​atc​tg-3′, rev: 5′-ggt​tcg​ctt​tct​ctt​tcg​gg-3′); *NANOG* (fw: 5′-acc​cag​ctg​tgt​gta​ctc​aa-3′, 5′-ctg​cgt​cac​acc​att​gct​at-3′); *SOX2* (fw: 5′-cca​aga​tgc​aca​act​cgg​ag-3′, rev: 5′-ggg​cag​cgt​gta​ctt​atc​ct-3′); *E-CAD* (fw: 5′-cgg​acg​atg​atg​tga​aca​cc-3′, 5′-ttg​ctg​ttg​tgc​tta​acc​cc-3′); *VIM* (fw: 5′-tgc​agg​ctc​aga​ttc​agg​aa-3′, 5′-ctc​cgg​tac​tca​gtg​gac​tc-3′); *SNAIL* (fw: 5′-atg​agg​aca​gtg​gga​aag​gc-3′, rev: 5′-gga​gat​cct​tgg​cct​cag​ag-3′); *SLUG* (fw: 5′-ctc​cat​ctg​aca​cct​cct​cc-3′, 5′-ttt​cta​gac​tgg​gca​tcg​ca-3′).

### 2.12 3D tumor spheroid invasion assay

To evaluate the invasive potential of HEY and PEO1 cells *in vitro*, 3D tumor spheroids generated from both cell lines were implanted into the Matrigel Matrix Basement Membrane (Corning Incorporated, United States). Briefly, a suspension of OVCA 3D tumor spheroids at very low density was seeded into a 96-well round-bottom ULA plate. Then, 100 µL of the medium was carefully removed, and 100 µL of Matrigel was added using ice-cold tips. The 96-well ULA plate was centrifuged at 300 *g* for 3 min at 4°C (to place spheroids in the center of each well) and the Matrigel was solidified through incubation at 37°C for 1 h. Then, 100 µL of complete cell culture medium was added on top of the spheroid containing Matrigel layer, and the initial spheroid size (0 h) was documented using Leica THUNDER Imaging Systems DMi8 (Leica Microsystems S.r.l., Wetzlar, Germany). In the same way, the invasion area was captured after 12, 24, 48 and 72 h of incubation. All measurements were analyzed using the internal measuring feature of Zen imaging software (Leica Camera AG, Wetzlar, Germany, Europe). The relative invasion area was finally determined by using ImageJ software (http://rsb.info.nih.gov/ij/). Each experiment was performed in triplicate.

### 2.13 Statistical analysis

Overall data are represented as mean ± standard deviation (SD) of at least three biological replicates. When appropriate, data were analyzed by performing a simple comparison between two groups using Student’s t-test. We were interested in determining whether the means of more than two groups were equal or not, thus, we performed an analysis of variance (ANOVA). A *p*-value < 0.05 was considered statistically significant.

## 3 Results

### 3.1 The generation of HEY and PEO1 tumor spheroids in non-adherent culture conditions is accompanied by the reduction of intracellular free-redox active iron

**FIGURE 1:**
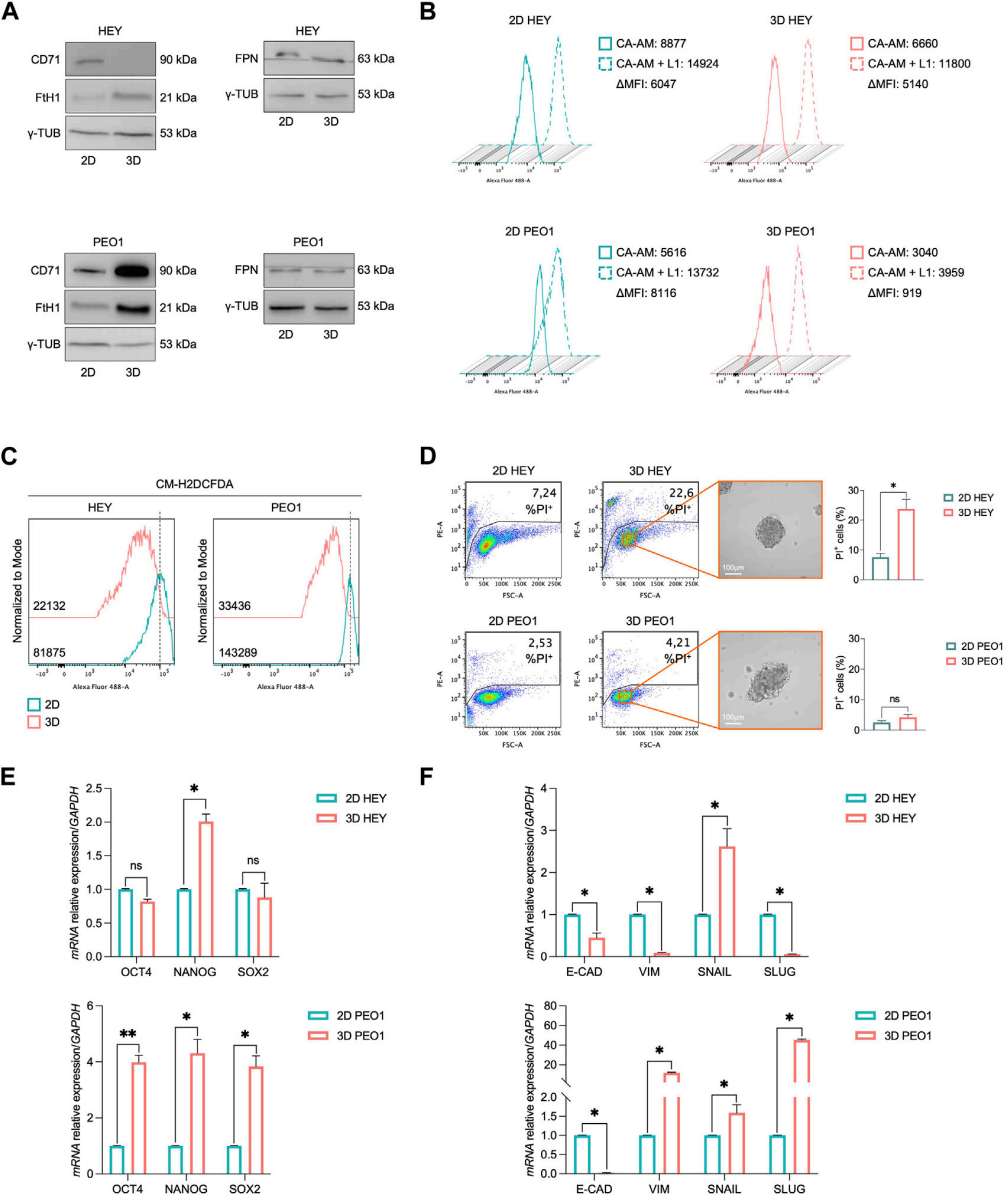
3D tumor spheroids derived from HEY and PEO1 cells show a reduction of intracellular LIP. **(A)** Western blot of FtH1, CD71, and FPN in HEY and PEO1 cells (3D vs. 2D). γ-TUB was used as loading control. Flow cytometry analysis of LIP **(B)** and ROS **(C)** amounts quantified by using CA-AM and CM-H2DCFDA respectively, in HEY and PEO1 cells cultured in both 2D, and 3D condition. **(D)** PI flow cytometric analysis of HEY and PEO1 cells (3D vs. 2D); % of dead cells (PI positive) are reported in each plot. Representative images of their derived 3D tumor spheroids (Scale bar: 100 μm; Magnification: ×20) and relative histogram of the % of dead cells are reported on the right. qRT-PCR of stemness **(E)** and EMT **(F)** markers in HEY and PEO1 cells (3D vs. 2D). Results are presented as mean ± SD from three independent experiments. *p-value <0.05; **p-value <0.01; ns: not significant.

Here, we analyzed the iron metabolism of PEO1 and HEY ovarian cancer cell lines during the transition from adherent (2D) to non-adherent (3D) culture conditions. We found that in both the cell lines, this process is accompanied by the decrease of free and redox-active iron pool and the increase of the main iron storage protein subunit FtH1 (Figures 1A, B). The extent of FtH1 upregulation, though, is significantly different between the two cell lines, being of the order of 2.5-fold in HEY 3D spheroids and of 8.4-fold in PEO1-derived 3D spheroids (Supplementary Figure S1). In agreement, the amount of free labile iron pool (LIP) is reduced of about 9-fold in PEO1 3D tumor spheroids compared to the relative 2D cultures (CA-AM, PEO1 2D: 8,116 vs. PEO1 3D: 919); in HEY 3D spheres, instead, the LIP is only slightly decreased (CA-AM, HEY 2D: 6,047 vs. HEY 3D: 5,140) (Figure 1B). Interestingly, the quantification of intracellular oxidative stress highlighted a reduction of ROS in both 3D HEY and 3D PEO1 compared to their relative 2D counterparts, with a more pronounced reduction in PEO1 tumor spheroids (DCF, PEO1 2D: 143,289 vs. PEO1 3D: 33,436; HEY 2D: 81,875 vs. HEY 3D: 22,132) (Figure 1C). The control of iron intake appears opposite between the two cell lines during 2D to 3D transition. PEO1 tumor spheroids foster iron uptake, as demonstrated by the upregulation of CD71 protein levels, while HEY tumor spheroids shut the receptor down. Concerning the iron efflux, FPN protein levels remain substantially unaltered both in HEY and PEO1 cells during the transition from 2D to 3D (Figure 1A). To rule out the possibility that different media, used to grow HEY and PEO1 cells in 2D and 3D culture conditions, might influence the iron status we checked for the baseline concentration of iron within the different culture media. In DMEM and RPMI, used for HEY and PEO1 2D cultures respectively, iron is present only as trace element (0,0001 g/L Fe(NO_3_)_3_). The DMEM/F12 medium, instead, contains 0,00005 g/L of Fe(NO_3_)_3_ and 0,000,417 g/L of FeSO_4_. Considering that DMEM/F12 has been used to grow both HEY and PEO1 3D cultures, the above-described differences in the modulation of iron metabolism between HEY and PEO1, during the transition from 2D to 3D cultures, seem independent from the baseline concentration of iron within the different culture media.

The differences in iron handling are somewhat accompanied by a different viability between the two cell lines in the 3D culture conditions. Around 22.6% of HEY tumor spheroids face cell death while PEO1 cells appear totally unaffected (Figure 1D). PEO1-derived tumor spheroids appear also acquiring a typical CSC-like and an EMT-like phenotype characterized by a significant overexpression of *Oct4*, *Nanog*, and *Sox2* stemness marker genes and a strong upregulation of the mesenchymal marker Vimentin (*Vim*) and its transcription factors *Snail* and *Slug* and a parallel breakdown of the epithelial marker e-cadherin (*E-cad*). In HEY-derived tumor spheroids, instead, only *Nanog* and *Snail* appear increased and *E-Cad* results halved. *Vim* and *Slug* modulation is not properly consistent with an EMT process (Figures 1E, F). Overall, these results suggest that the ovarian cancer cell growth in non-adherent culture conditions requires a modification of iron metabolism essentially aimed at preventing the accumulation of free and redox-active iron. Between the two cell types, though, PEO1 spheroids show a more pronounced capacity to buffer the intracellular LIP by markedly up-regulating FtH1 levels. This property is accompanied by higher viability and a more pronounced CSC-like phenotype of PEO1-derived tumor spheroids.

### 3.2 Excess environmental iron impairs expansion and invasive properties of tumor spheroids derived from HEY but not from PEO1 cells

**FIGURE 2:**
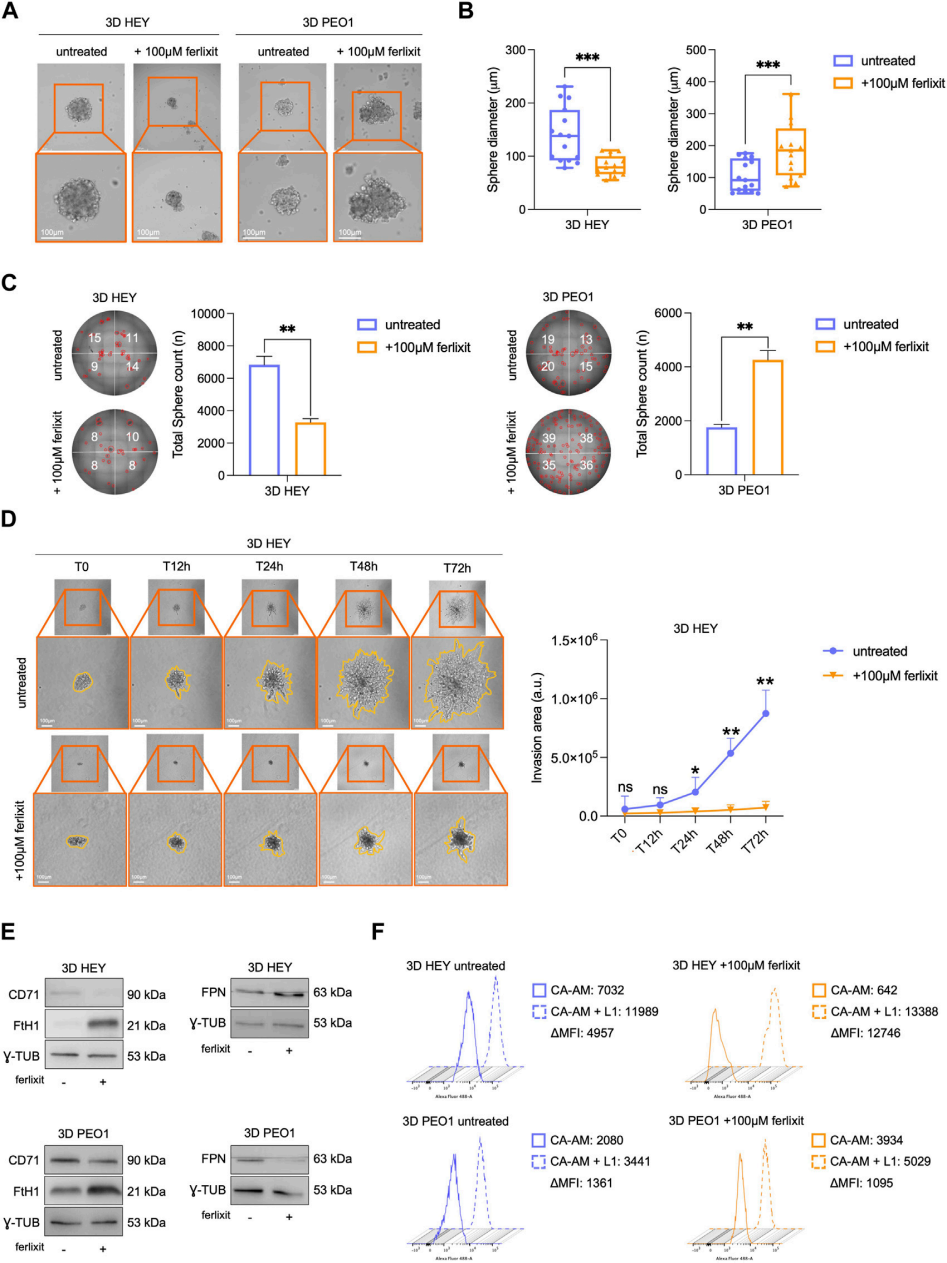
Ferlixit administration impairs 3D tumor spheroid formation and relative invasive ability only in HEY cells. Representative images and relative histograms of 3D HEY and PEO1 tumor spheroids morphology **(A)**, diameter **(B)** and count **(C)** upon treatment with 100 μM ferlixit (Scale bar: 100 μm; Magnification: ×20). **(D)** Representative images and relative histograms of the invasion ability of 3D HEY tumor spheroid treated with 100 μM ferlixit or left untreated (T0, T12h, T24h, T48 h and T72 h). **(E)** Western blot of FtH1, CD71and FPN in 3D tumor spheroids derived from HEY and PEO1 cells untreated and treated with 100 μM ferlixit. γ-TUB was used as loading control. **(F)** Flow cytometry analysis of LIP amounts quantified by using CA-AM in 3D HEY and PEO1 tumor spheroids after administration of 100 μM ferlixit. All the experiments were carried out in triplicate and results are presented as mean ± SD. *p-value <0.05; **p-value <0.01; ***p-value <0.001; ns: not significant.

The observation that both HEY and PEO1 cells attenuate the levels of free redox-active iron during the transition from 2D to 3D culture conditions prompted us to assess whether supplementation with excess iron (100 μM Fe^3+^, ferlixit) within culture media could be sufficient to inhibit tumor spheroids generation. Of interest, we found that excess iron significantly reduces both the number (from 6,840 to 3,280, **p-value <0.01) and the size (from 139.8 μm to 82.2 μm, ***p-value <0.001) of tumor spheroids generated from HEY cells. In PEO1 cells, instead, excess iron does not attenuate tumor spheroid generation but, rather, enhances tumor spheroids number (from 1760 to 4,260, **p-value <0.01) and the size (from 105 μm to 184 μm, ***p-value <0.001) (Figures 2A–C). The analysis of the metastatic potential of HEY- and PEO1-derived tumor spheroids, performed by monitoring and quantifying invading spheroid area over time (12-24-48–72 h), highlighted that excess iron also triggers a marked inhibition of HEY spheroids invasion (time-course images and plot in Figure 2D and relative time-lapse in Movie 1–2). In PEO1 tumor spheroids, lacking invasive properties in normal iron culture conditions, supplementation with Fe^3+^ is not accompanied with any variation (Supplementary Figure S2 and relative time-lapse in Movie 3–4).

Concerning the regulation of iron metabolism in iron-rich culture media, HEY tumor spheroids continue to increase FtH1, further reduce CD71, and also increase FPN expression levels. PEO1 tumor spheroids, instead, maintain approximately unaltered CD71, reduce FPN while further strongly increase FtH1 (Figure 2E). Concerning the intracellular free iron levels, HEY tumor spheroids cultured in iron-rich media appear unable to store it (CA-AM, HEY 3D: 4,957 vs. HEY 3D 100 μM Fe^3+^: 12,746). In PEO1 tumor spheroids, instead, the intracellular LIP shows a slight decrease, thus suggesting a remarkable storage ability (Figure 2F). Overall, these results suggest that an iron-rich environment may cause the accumulation of free redox-active iron which in turn, can inhibit the sphere-forming ability of ovarian cancer cells in ECM-detached conditions. This phenomenon is, though, cell type dependent.

### 3.3 Excess environmental iron causes ferroptosis in HEY tumor spheroids

**FIGURE 3:**
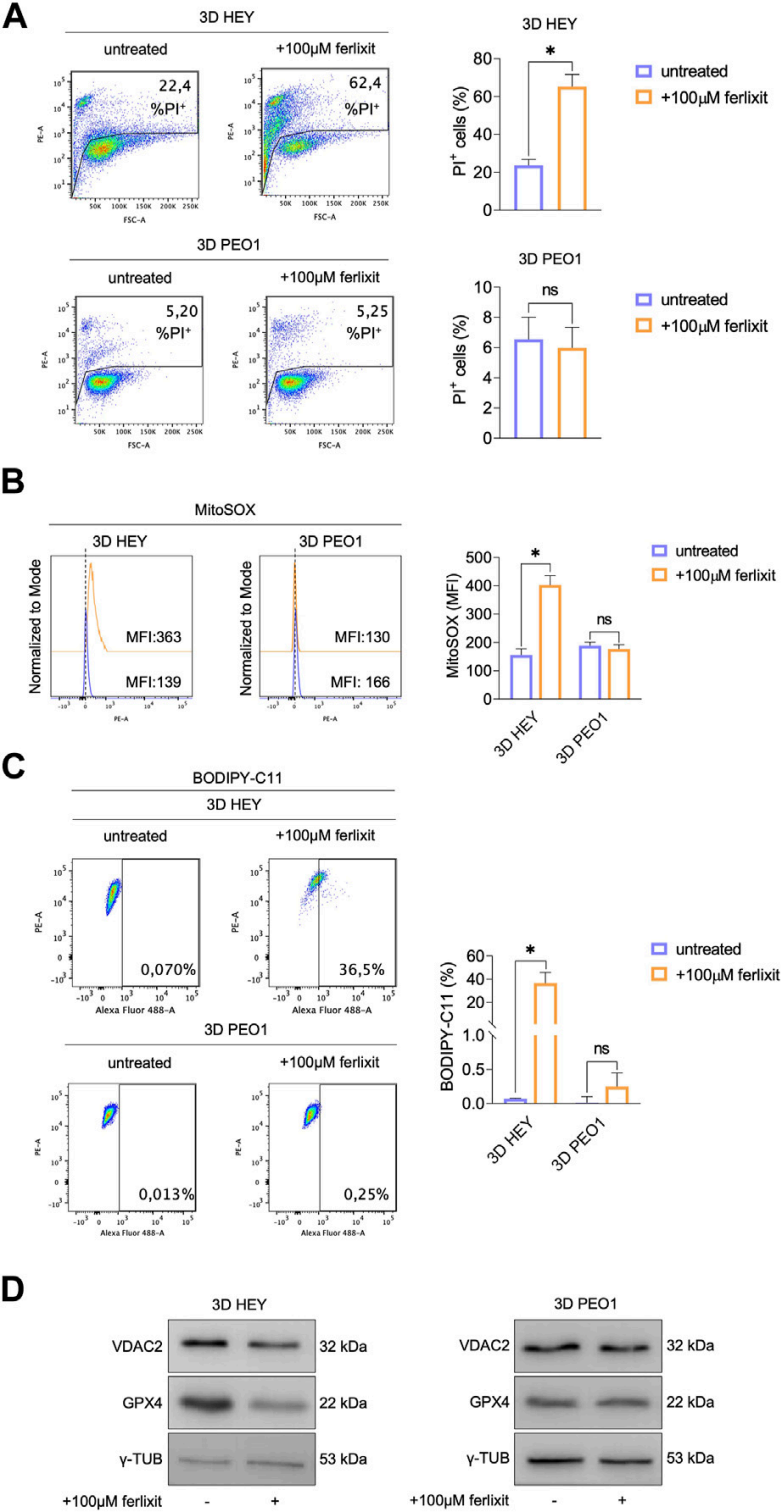
Treatment with ferlixit leads to ferroptosis in 3D HEY tumor spheroids. **(A)** PI flow cytometric analysis of 3D HEY and PEO1 tumor spheroids untreated and treated with 100 μM ferlixit. % of dead cells (PI positive) are reported in each plot. Relative histogram is reported on the right. Flow cytometry analysis of mitochondrial ROS levels **(B)** and lipid peroxidation **(C)** quantified by using MitoSOX and BODIPY-C11 reagent, respectively, in 3D HEY and PEO1 tumor spheroids after administration of 100 μM ferlixit. Relative histograms are reported on the right. **(D)** Western blot of VDAC2 and GPX4 in 3D HEY and PEO1 cells upon administration of 100 μM ferlixit. γ-TUB was used as loading control. Results were obtained from three independent experiments.

The accumulation of intracellular free redox-active may trigger tumor cell death through ferroptosis (Battaglia et al., 2020). Here, by means of PI flow cytometry analysis, we found that supplementation with 100 μM Fe^3+^ enhances mortality of HEY tumor spheroids up to 62.4% while leaving that of PEO1 tumor spheroids at 5.25% (Figure 3A). The quantification of mitochondrial ROS (MitoROS) and lipid peroxidation, the two main biochemical features of ferroptosis, show that iron supplementation causes a 2-fold increase of mitoROS (MitoSOX, HEY 3D: 139 vs. HEY 3D 100 μM Fe^3+^: 363) and the accumulation of lipid peroxides up to 36.5% of HEY tumor spheroids (Figures 3B, C). In agreement, the ferroptosis markers GPX4 and VDAC2 result significantly decreased (Figure 3D). Of note, none of the biochemical signs of ferroptosis have been detected in PEO1 tumor spheroids.

Collectively, these data indicate that the survival in non-adherent culture conditions of ovarian cancer cells might depend on ferroptosis sensitivity.

### 3.4 *FtH1* knockdown impairs sphere-forming ability of PEO1 cells

**FIGURE 4:**
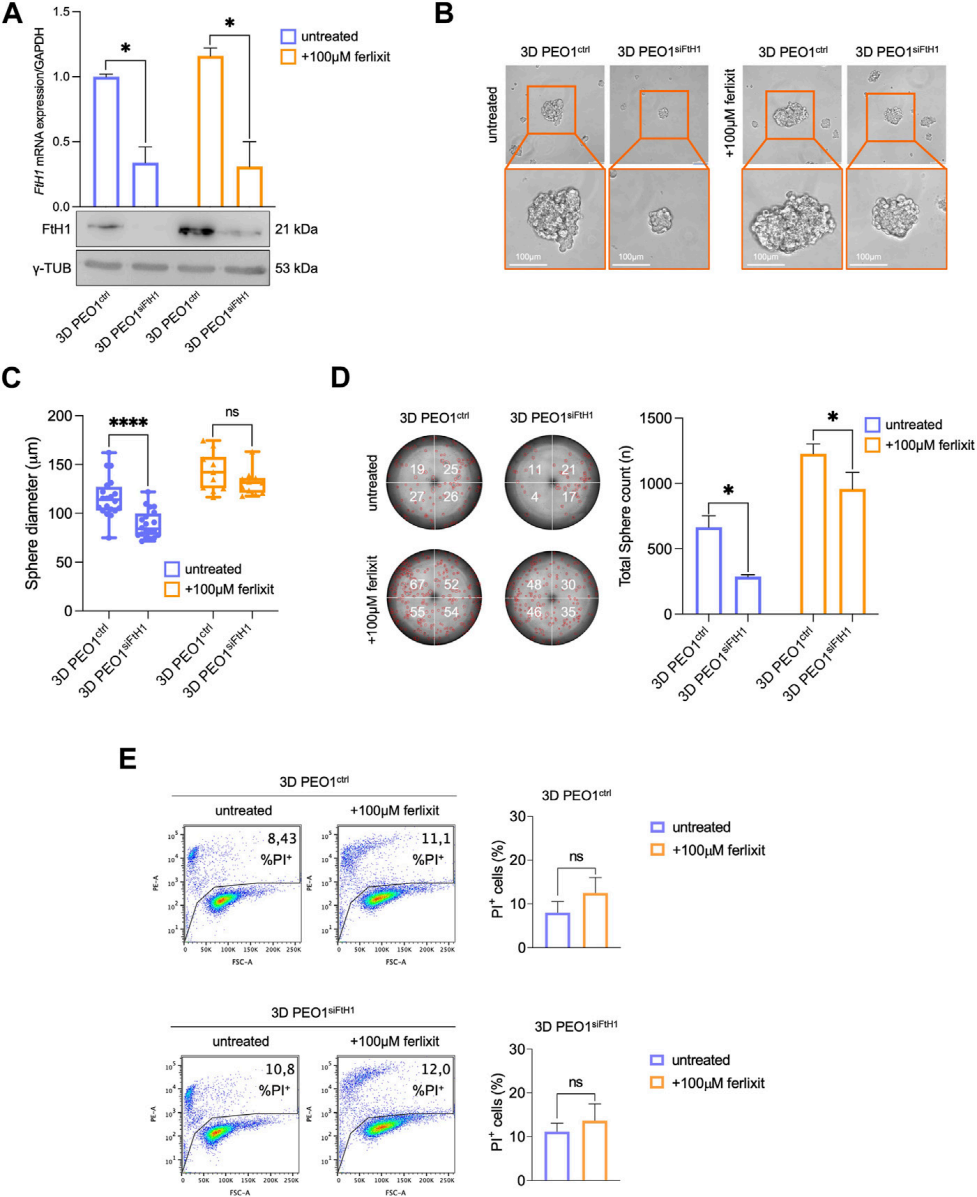
*FtH1* silencing reduces the ability to form 3D tumor spheroids in PEO1 cells. **(A)** qRT-PCR and WB analysis of FtH1 in 3D PEO1 tumor spheroids upon FtH1 silencing. Representative images and relative histograms of 3D PEO1 tumor spheroids morphology **(B)**, diameter **(C)** and count **(D)** after FtH1 knockdown (Scale bar: 100 μm; Magnification: ×20). **(E)** PI flow cytometric analysis of 3D PEO1^ctrl^ and 3D PEO1^siFtH1^.% of dead cells (PI positive) are reported in each plot. Relative histogram is reported on the right. Results are presented as mean ± SD from three independent experiments. *p-value <0.05; ****p-value <0.0001; ns: not significant.

In light of the results described above, we finally assess the contribution of elevated FtH1 levels to the ability of PEO1 to survive and generate tumor spheroids in ECM-detached conditions both in iron-rich and non-iron-rich environmental conditions. To this, we performed the transient knockdown of FtH1 and upon 4 days we observed a reduction of FtH1 gene expression of about 70% in PEO1 tumor spheroids grown both in iron-rich and non-iron rich culture media (Figure 4A). Notably, we found that siRNA targeting FtH1 is sufficient to significantly impair the sphere-forming ability of PEO1 cells. When grown in a non-iron rich culture medium, FtH1 knockdown causes a significant reduction of both number (from 664 to 286, *p-value <0.05) and size (from 116.1 μm to 88.8 μm, ****p-value <0.0001) of tumor spheroids. When grown in the culture medium supplied with iron, FtH1 silencing remarkably diminishes the number of tumor spheroids (from 1,228 to 958, *p-value <0.05) without affecting their size (Figures 4B–D). The flow cytometry analysis of cell death, instead, showed that FtH1 knockdown is unable to promote cell mortality in neither of the two culture media (Figure 4E). Taken together, these findings suggest that when the FtH1 upregulation associated with the 2D to 3D transition is prevented, PEO1 cells significantly reduce their ability to generate tumor spheroids. However, this is not accompanied by an increase of ferroptosis sensitivity.

## 4 Discussion

Ovarian cancer shows a high metastatic potential (Motohara et al., 2018). Tumor cells detach from the ECM and either disseminate into the peritoneal cavity or migrate via the bloodstream and the omentum (Rakina et al., 2022). As such, ovarian cancer is an aggressive disease with a very poor prognosis (Oliveira et al., 2021). In the last years, a plethora of scientific researches have provided compelling evidence denoting a close association between initiation, progression, and metastasis of ovarian cancer with dysregulation of iron homeostasis (Rockfield et al., 2017; Brown et al., 2020; Lelièvre et al., 2020). Indeed, starting from the early stages of cancer development, ovarian tumor cells acquire an “iron-addicted phenotype” characterized by enhanced iron uptake and retention, as a consequence of increase of the iron importer CD71, decrease of the iron efflux pump FPN, and increase of the iron storage protein FtH1 (Walsh et al., 2009). Iron addiction appears even more pronounced in the subset of CSCs, responsible of both tumor initiation and metastasis (Cosialls et al., 2021). Here, iron is not only required for proliferation and cell cycle progression (Le and Richardson, 2002), but also in the production and release of intereluikin-6 (IL-6), which in turn promotes the upregulation of MMPs and the tumor invasion (Sacco et al., 2021). However, if iron homeostasis is disrupted, free iron is engaged in Fenton reactions-mediated production of ROS, thus leading to ferroptosis. In this regard, we and others have demonstrated that the activation of ferritinophagic process, i.e., the degradation of ferritin shell, mediated by nuclear co-activator 4 (NCOA4), and the consequent release of free iron into the cytosol, primarily contributes to ferroptosis occurrence in ovarian cancer cells (Ying et al., 2021; Battaglia et al., 2022).

Fundamental contributions to understanding the role of iron metabolism in the detachment from ECM arises mainly from two recent studies (Wang et al., 2021; He et al., 2023). In 2021, Wang, Q. et al. found that the detachment of ovarian cancer cells from the ECM is associated with the increase of LIP. In particular, by using *in vitro* models, the authors demonstrated that ovarian tumor spheroids show increased iron level compared to their relative adherent cultures and that this was mainly due to the increase of iron uptake mediated by both the divalent metal transporter 1 (DMT1) and CD71. Moreover, Wang, Q. et al. found that, to use iron efficiently, ovarian cancer spheroids also enhance FtH1 and the iron chaperon Poly(RC) Binding Proetin 1 (PCBP1) (Wang et al., 2021). Diversely, later in 2023, He, J., et al. found that to survive in detached conditions, cancer cells maintain the intracellular LIP at low levels through the increase of FtH1-mediated iron storage in association with the reduction of iron intake protein CD71 (He et al., 2023). According to this research group, this is a general mechanism of self-protection during the ECM detachment and extends across multiple cancer types.

In our study, we explored the effect of the environmental iron abundance in the ability of HEY and PEO1 ovarian cancer cells to survive and grow in detached culture conditions. In agreement with the two researches from Wang, Q. et al. and He, J. et al., our results show that, to generate 3D tumor spheroids, both HEY and PEO1 cells enhance the iron storage capacity by upregulating the iron storage protein FtH1, although at different extent. Indeed, PEO1 3D tumor spheroids strongly upregulate FtH1 while HEY 3D tumor spheroids only moderately increase it. Notably, our findings also highlight some significative differences compared to the two previous studies. Diversely from Wang, Q. et al., we demonstrate that during the 2D to 3D transition, HEY and PEO1 reduce the intracellular free and redox-active iron (LIP). We believe that this should not necessarily be considered a conflicting result, as Wang. Q, et al. showed an increase of iron uptake and LIP at 12 h of 3D culture conditions followed by a drop down through 24h–72 h. In our study, we have measured LIP amount only upon 96 h of 3D tumor spheroid generation. Concerning the control of iron uptake, we observed that HEY and PEO1 tumor spheroids act in a very opposite way. Indeed, while PEO1 spheroids strongly enhance CD71, HEY spheroids shut down it. No differences were, instead, observed in the control of iron efflux mediated by FPN. Taken all together, our results suggest that to grow in detached-culture conditions, PEO1 cells show a higher iron demand and, also, a more pronounced capacity to handle iron storage and to avoid the intracellular accumulation of free redox-active iron compared to HEY cells. Indeed, in HEY spheroids LIP is slightly decreased while in PEO1 spheroids LIP is significantly diminished. In addition, intracellular ROS drop down during the 2D to 3D transitions in both the cell lines, but more significantly in PEO1 cells. The hypothesis of a different iron addiction between the two cell lines is supported by two main observations. First, PEO1 cells, already at baseline 2D culture conditions, show an expression pattern of the iron regulatory proteins (i.e., higher CD7, higher FtH1, and lower FPN) that is typical of a pronounced iron import and retention. Second, upon excess iron supplementation within the surrounding environment, PEO1 cells continue to enhance iron intake and iron storage and continue to decrease FPN without accumulating the intracellular and potential damaging LIP. As a consequence, PEO1 cells do not undergo oxidative stress and relative ferroptosis, but rather generate a greater number of larger tumor spheroids. HEY cells grown in an iron-rich environment, instead, significantly attenuate their sphere-forming ability and invasiveness; what’s more, they undergo strong lipid peroxidation and mitochondrial dysfunction, which ultimately lead to ferroptosis.

The different behavior of HEY and PEO1 cells grown in an iron-rich environment is a main difference with He J et al. Indeed, they demonstrated that either iron supplementation, or treatment with ferroptosis inducers (i.e., erastin and RSL3), or the genetic manipulation of ferroptosis modulators (i.e., GPX4) are cytotoxic in all cancer cell types during the detachment from ECM (He et al., 2023). Taken all together, we believe that suggest that enhanced levels of iron are required for ovarian cancer growth and metastasis but, at the same time, they might represent an Achilles heel potentially exploitable for cancer therapy. Of note, not all cell types equally respond to this input but, rather, diverse ovarian cancer cells display different susceptibility to ferroptosis. Our data, instead, raise the distinct possibility that, also between different cells belonging to the same tumor type, a diverse inherited control of iron metabolism and a different inherited iron demand may account for a diverse ferroptosis sensitivity, which remarkably affects the success of the ECM-detachment. Therefore, we finally tried to limit the iron storage capacity and the antioxidant defense of PEO1 cells by performing *FtH1* knockdown. Notably, we found that *FtH1* silencing is sufficient to reduce the generation and expansion of tumor spheroids; however, it is unable to sensitize PEO1 cells to ferroptosis, and cell viability results untouched. The combined manipulation of other endogenous antioxidant enzymes will be important to better understand whether this approach would be useful to overcome ferroptosis resistance and, thus, kill all ECM-detached ovarian cancer cells.

Over the past 5 years, treating ovarian cancer cells with agents that induce the iron-dependent ferroptosis has emerged as a new strategy for turning the concept of “iron addiction” to a therapeutic advantage. In this study, we demonstrate that the reprogramming of iron metabolism is a key event during ECM detachment that could be targeted to specifically eliminate metastatic ovarian cancer cells. However, sensitivity to iron-dependent ferroptotic cell death significantly varies not only across different cancer types but also across multiple cells within the same tumor mass. Thus, understanding the sensitivity (or insensitivity) of ECM-detached ovarian cancer cells to ferroptosis-inducing treatments is mandatory not only to generate important new biological insight on the mechanisms underlying ovarian cancer metastasis, but also to develop more effective approaches to treat this highly metastatic disease.

## Data Availability

The original contributions presented in the study are included in the article/Supplementary Material, further inquiries can be directed to the corresponding author.
