# Supplementary material for: Iron affects the sphere-forming ability of ovarian cancer cells in non-adherent culture conditions
Source: Front Cell Dev Biol. 2023 Nov 14;11:1272667. doi: 10.3389/fcell.2023.1272667 (PMC10682100; doi:10.3389/fcell.2023.1272667)
Supplement: Supplementary file 5 [file DataSheet1.DOCX]

Supplementary Material

Iron affects the sphere-forming ability of ovarian cancer cells in non-adherent culture conditions

Anna Martina Battaglia^†1^, Alessandro Sacco^†1^, Eleonora Vecchio^1^, Stefania Scicchitano^1^, Lavinia Petriaggi^1^, Emanuele Giorgio^1^, Stefania Bulotta^2^, Sonia Levi^3^, Concetta Maria Faniello^1^, Flavia Biamonte*^1,4^, Francesco Costanzo^1,4^

*** Correspondence:** Flavia Biamonte: flavia.biamonte@unicz.it

## Supplementary Figures


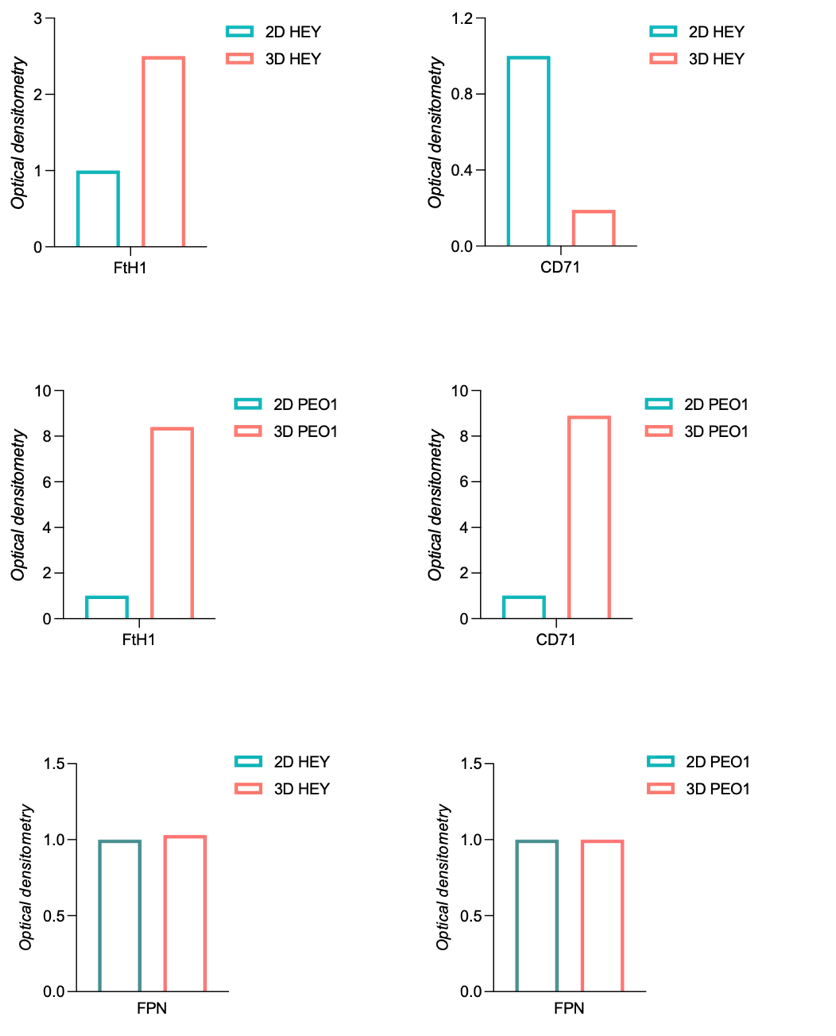


**Supplementary Figure 1.** Optical densitometry relative to western blot analysis of FtH1, CD71 and FPN in HEY and PEO1 cells (3D vs 2D) by using ImageJ sofware.


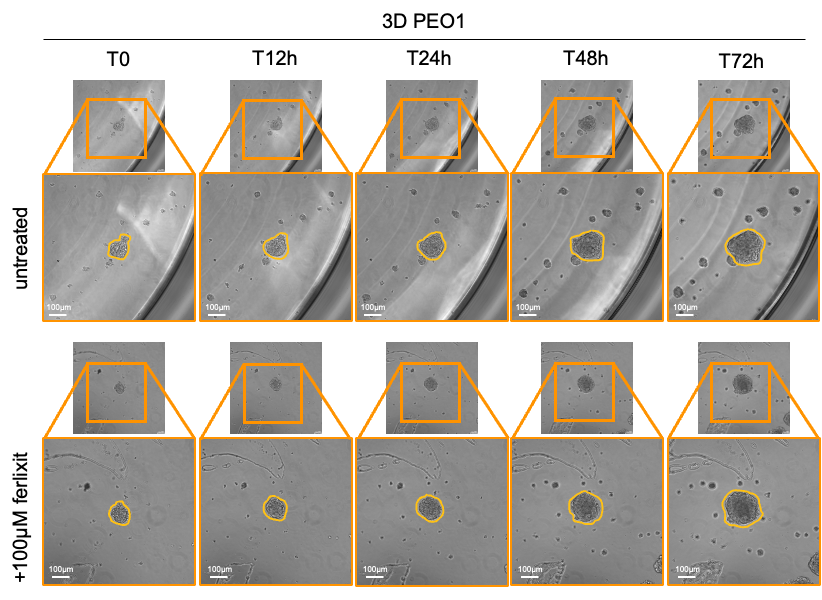


**Supplementary Figure 2.** Representative images of the invasion ability of 3D PEO1 tumor spheroid treated with 100μM ferlixit or left untreated (T0, T12h, T24h, T48h and T72h). (Scale bar: 100μm; Magnification: 20x).
